# Current clinico-mycological trends of onychopathies in Casablanca

**DOI:** 10.1186/1471-2334-14-S2-P97

**Published:** 2014-05-23

**Authors:** L Badaoui, G Dabo, R Bensghir, I Halim, S Belyamani, M Soussi Abdallaoui, S Chiheb, K El Filali Marhoum

**Affiliations:** 1 Ibn Rochd University Hospital, Casablanca, Morocco

## Background

Onychopathies in patients infected with HIV are dominated by onychomycosis. It is one of the early manifestations of HIV infection .the purpose was to study the epidemiology, clinical manifestations and mycological profile of onychomycosis in HIV-infected individuals and to identify the other causes of onychopathies in this population.

## Methods

A total of 88 HIV infected patients, monitoring at Department of Infectious Diseases, were screened for nail involvement over a period of 3 months. Inclusion criteria: Patient who had do not receive any antifungal treatment were included.

## Results

A total of 88 subjects were examined (45men and 43 women); the average age were 41 years old. Abnormal appearing nails were present in 48 cases. Mycologic evidence of onychomycosis were present in 42 cases. The clinical presentation of onychomycosis was: distal and lateral subungual onychomycosis (47%); white superficial onychomycosis (5%); proximal subungual onychomycosis (21%); total dystrophic onychomycosis (26%). Toenail involvement was seen in 26 patients, fingernail in 11 patients while 5 patients had involvement of both fingernail and toenail. The various aspects noted were: Xanthonychia (19 cases), pachonychia (17 cases), onycholysis (7 cases), leuconychia (6 cases) and melanonychia (4 cases). The culture has allowed the confirmation of onychomycosis in 73.3%. Among the 16 positive dermatophytes cultures, Trichophyton rubrum was isolated in 12 cultures, Trichophyton interdigitale in 3 cultures, and Trichophyton Mentagrophyte in one case. Of the 12 non-dermatophytic cultures, Candida albicans and Candida dubliensis were isolated in 4, Candida parapilosis in 2 while Trichosporon spp., and Penicillium spp. in each one.

## Conclusion

The prevalence of onychomycosis was 48%. It seems that the incidence of NMD is higher in the HIV patients compared with immunocompetent population.

